# Thiamine supplementation holds neurocognitive benefits for breastfed infants during the first year of life

**DOI:** 10.1111/nyas.14610

**Published:** 2021-06-07

**Authors:** Jeffrey R. Measelle, Dare A. Baldwin, Jelisa Gallant, Kathleen Chan, Tim J. Green, Frank T. Wieringa, Mam Borath, Sophonneary Prak, Daniela Hampel, Setareh Shahab‐Ferdows, Lindsay H. Allen, Hou Kroeun, Kyly C. Whitfield

**Affiliations:** ^1^ Department of Psychology University of Oregon Eugene Oregon; ^2^ Department of Applied Human Nutrition Mount Saint Vincent University Halifax Nova Scotia Canada; ^3^ South Australian Health and Medical Research Institute Adelaide South Australia Australia; ^4^ UMR‐204 Institut de recherche pour le développement Montpellier France; ^5^ National Sub‐Committee for Food Fortification Cambodia Ministry of Planning Phnom Penh Cambodia; ^6^ National Nutrition Programme, Maternal and Child Health Centre Cambodia Ministry of Health Phnom Penh Cambodia; ^7^ USDA/ARS Western Human Nutrition Research Center Davis California; ^8^ Department of Nutrition University of California Davis California; ^9^ Helen Keller International Cambodia Phnom Penh Cambodia

**Keywords:** thiamine (vitamin B_1_), supplementation, Cambodia, infancy, cognitive development

## Abstract

Women reliant on mostly rice‐based diets can have inadequate thiamine intake, placing breastfed infants at risk of thiamine deficiency and, in turn, physical and cognitive impairments. We investigated the impact of maternal thiamine supplementation doses on infants’ cognitive, motor, and language development across the first year. In this double‐blind, four‐parallel‐arm, randomized controlled trial, healthy mothers of exclusively breastfed newborn infants were recruited in Kampong Thom, Cambodia. At 2 weeks postnatal, women (*n* = 335) were randomized to one of four treatment groups to consume one capsule/day with varying amounts of thiamine for 22 weeks: 0, 1.2, 2.4, and 10 mg. At 2, 12, 24, and 52 weeks of age, infants were assessed with the Mullen Scales of Early Learning (MSEL) and the Caregiver Reported Early Development Instrument (CREDI). Multiple regression and mixed effects modeling suggest that by 6 months of age, the highest maternal thiamine dose (10 mg/day) held significant benefits for infants’ language development, but generally not for motor or visual reception development. Despite having achieved standardized scores on the MSEL that approximated U.S. norms by 6 months, infants showed a significant drop relative to these norms in both language domains following trial completion, indicating that nutritional interventions beyond 6 months may be necessary.

## Graphical abstract

Women reliant on mostly rice‐based diets can have inadequate thiamine intake, placing breastfed infants at risk of thiamine deficiency and, in turn, physical and cognitive impairments. We investigated the impact of maternal thiamine supplementation doses on infants’ cognitive, motor, and language development across the first year in a double‐blind, randomized controlled trial in Cambodia that recruited healthy mothers of exclusively breastfed newborn infants.

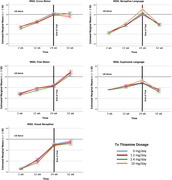

## Introduction

In 2003, infant formula erroneously manufactured without thiamine was sold in Israel.^1^ The public and personal health consequences were significant as prolonged consumption resulted in a number of infants suffering serious, long‐lasting health consequences. In addition, affected infants who displayed no obvious clinical signs of thiamine deficiency, nevertheless, experienced long‐term consequences.^2^ Among other health outcomes, many of these infants subsequently displayed measurable cognitive and motor delays, in particular, impaired language development.^1^, ^3^, ^4^, ^5^

These findings raised widespread concern because millions of infants, especially in regions, such as South and Southeast Asia, currently remain at high risk of thiamine deficiency due to low maternal thiamine intake during lactation,^6^, ^7^ and may thus also be vulnerable to compromised cognitive and language functioning. Discovering how best to protect the health and well‐being of infants who are developing under these circumstances is, therefore, an important public health goal and the basis of the larger clinical trial from which this report derives.^8^ Our focus in the present study was to determine the extent to which thiamine supplementation of lactating, rural Cambodian mothers, who are among those at risk of thiamine deficiency, might protect their breastfed infants’ developmental progress, with particular attention to cognitive, motor, and language development.

Neurocognitive development begins prenatally and then progresses rapidly from birth onward. Nutrient requirements to support not only growth but also the rapid and energy‐intensive neurocognitive change during this early period of life are particularly high.^9^ As a result, nutritional deficiencies—including thiamine deficiency—can undercut development in foundational and even irreversible ways that have cascading negative consequences for ultimate sociocognitive functioning.^10^, ^11^ Neurocognitive outcomes, therefore, must be measured directly during infancy using validated assessments to ascertain maternal thiamine biomarker levels sufficient to protect infants’ developmental well‐being.

### Study objectives

The present study was part of a larger, preregistered double‐blind, four‐parallel‐arm, randomized controlled trial monitoring the impact of daily maternal thiamine supplementation between 2 and 24 weeks postnatal on the thiamine status of both mothers and infants,^8^ with an additional follow‐up at 52 weeks. Our specific focus in this study was to examine the extent to which early thiamine exposure might be associated with improvements in infants’ early cognitive development. These cognitive outcomes were considered secondary outcomes at the time of preregistration.

### Hypotheses

The overarching hypothesis was that higher dosage levels of maternal thiamine supplementation would be associated with improved infant cognitive outcomes; specifically, enhanced motor, perceptual, and language development by the end of the trial. Based on the earlier work of Fattal‐Valvesk and colleagues,^1^, ^3^, ^4^ we hypothesized that supplementation effects would be strongest in the language domain. Analyses focused specifically on addressing the following questions (see Ref. 8 for details):

1. To what extent was breastfeeding mothers’ daily thiamine supplementation dose (0, 1.2, 2.4, or 10 mg) positively associated with infants’ cognitive outcomes, as measured by the Mullen Scales of Early Learning (MSEL^12^) and the Caregiver Reported Early Development Instrument (CREDI^13^), at our primary outcome assessment at 24 weeks? We include identical analyses of outcomes before and following this primary time point (i.e., 12 and 52 weeks, respectively) but employed corrections for multiple testing.
2. To what extent were breastfeeding mothers’ 2‐week baseline milk total thiamine concentrations (when infants were just 2‐weeks old and thiamine supplementation had not yet begun) positively associated with infants’ cognitive outcomes at 24 weeks? This question was extended to our cognitive outcomes at 12 and 52 weeks, with corrections again applied for multiple testing.
3. To what extent did mothers’ thiamine supplementation dose alter the longitudinal trajectory of change in infants’ cognitive outcomes throughout and following the period of supplementation?

Although not a preregistered objective,^8^ we also provide descriptive comparisons of the Cambodian infants’ standardized cognitive scores in the present study with U.S. norms collected during the validation of the MSEL,^12^ as well as with age‐corrected standard scores used to norm the CREDI^13^ in a multinational study. Such comparisons will help in the detection of specific cognitive domains that may be particularly vulnerable to developmental disruption during critical periods of early development among infants from low‐ and middle‐income countries (LMICs) relative to infants from more affluent developmental contexts.

## Methods

### Study design

The full study protocol is published elsewhere.^8^ This community‐based, double‐blind, four‐parallel‐arm randomized controlled trial took place between September 2018 and December 2019 in Kampong Thom province, Cambodia.

### Participants

Women were recruited through antenatal care visits at eight health centers throughout Kampong Thom province, and entered into the study at 2 weeks postpartum. Full eligibility criteria have been published;^8^ briefly, participants were healthy mothers (18–45 years old) to healthy, singleton, exclusively breastfed newborns. Mothers were not currently participating in any nutrition programs beyond normal care, and had not consumed any thiamine‐containing supplements in the 4 preceding months. Routine perinatal medical care in Cambodia includes the provision of iron and folic acid supplements during pregnancy (90 tablets) and lactation (30 tablets). Women in our study received an average (SD) of 84 (25) and 32 (15) tablets in pregnancy and lactation, respectively. Ethical approval was obtained from the National Ethics Committee for Health Research, Cambodia (112/250NECHR); Mount Saint Vincent University Research Ethics Board, Canada (2017‐141); and the University of Oregon Institutional Review Board, USA (07052018.008). Women provided written informed consent for themselves and their infant.

### Randomization, compliance, and masking

Women were randomized at 2 weeks postpartum to one of four treatment groups (placebo: 0 mg; estimated average requirement (EAR): 1.2 mg; double the EAR: 2.4 mg; and a positive control group: 10 mg), and asked to consume one capsule daily between 2 and 24 weeks postpartum. These opaque capsules contained varying amounts of thiamine hydrochloride and cellulose filler, and were formulated, compounded, and packaged in identical 14‐day blister packs with uninformative printed alphanumeric treatment code labels at the Quinpool Wellness Centre in Halifax, Nova Scotia, Canada. Compliance was assessed fortnightly: research assistants visited the participant's home to collect the old blister pack and complete a capsule count, and to deliver a new blister pack. Women were considered compliant if they consumed ≥ 80% capsules over the 22‐week intervention. Compliance was high, with 89% of women consuming at least 80% of their capsules.^14^

A computer‐generated randomization schedule was prepared by the study statisticians using ralloc.ado in Stata (College Station, TX) with blinded treatment code labels. Randomly permuted blocks of size 8 within‐health‐center strata were used to assign participants to one of eight treatment codes in the ratio 1:1:1:1:1:1:1:1 (two treatment codes per treatment group to assist with blinding). The treatment group mapping to each treatment code was performed by an independent scientist. The treatment codes were kept in sealed opaque envelopes labeled with the study ID and opened by the research assistants when a participant was enrolled in the study. Participants, research assistants, and study investigators were blinded to the randomized groups. Data analysts were necessarily unblinded during the analysis due to the primary analysis.^8^

### Procedures

Participant demographic, socioeconomic, and health information were collected by healthcare workers at delivery, and by field workers at baseline (2 weeks postnatal), midline (12 weeks postnatal), endline (24 weeks postnatal), and at 1 year (52 weeks postnatal). Using calibrated instruments and standard protocols,^15^ infant anthropometric measurements (weight, length, and head circumference) were collected. Length‐for‐age Z scores were computed using the WHO Anthro plug‐in for SPSS.

Standardized tests of infant neurological and cognitive development were collected by field staff at all four time points. Several mother–infant interaction tasks were also conducted and video recorded for later analysis at all time points; these interaction tasks are not reported on in the present study.

Human milk samples were collected at 2, 4, 12, and 24 weeks postpartum using a battery‐powered single‐breast pump (Swing Breast pump, Medela) in women's homes or a central village meeting space. One full, single‐breast expression was collected from the breast that participants self‐identified as “fuller.” Samples were transported to the field lab in Kampong Thom in iceboxes within 5 h of collection. All milk samples were stored at −20 °C for up to 10 days, then moved to −80 °C before subsequent batch shipping to labs on dry ice for analysis.

Human milk thiamine concentrations were assessed^16^ at the U.S. Department of Agriculture ARS Western Human Nutrition Research Center (Davis, CA) as previously published, with a few changes: only 100 μL of milk was used, and deoxypyridine was added as an internal standard. After precolumn thiochrome derivatization of the target analytes, the sample extracts were measured using an Agilent 1200 HPLC with fluorescence detector. Milk total thiamine concentrations used here were calculated based on molecular weights: total thiamine = free thiamine + (thiamine monophosphate × 0.871) + (thiamine diphosphate × 0.707).

### Cognitive measures

#### Standardized test of early development

Child development was assessed using an adapted version of the MSEL^12^ administered to infants at all four time points by trained field staff. The MSEL is a standardized developmental test for children between birth to 68 months old; performance‐based items assess child outcomes in five developmental domains: gross motor, fine motor, visual reception, receptive language, and expressive language. The MSEL has previously been used in other LMICs, and our staff adapted the protocol by substituting potentially unfamiliar images, objects, and questions with objects and examples that Cambodian infants would recognize (following procedures recommended by Peña^17^). No modifications to the MSEL's test stimuli or administration procedures were needed at any of the assessment ages; however, there were slight variations in the actual toys used to test different abilities (e.g., crackers substituted for Cheerios and box with a coin slot for a piggy bank). Because to our knowledge this was the first use of the MSEL with Cambodian infants, we piloted our translated version of the MSEL with approximately 10 infants before each assessment to increase confidence in its test validity.

Each subscale consists of a set of performance‐based items (typically 0 for failed and 1 for passed), presented in hierarchical order of difficulty, where children are rated on whether they successfully complete task items to establish basal (three consecutive passed items) and ceiling (discontinuity after three consecutive unpassed items) ranges with which to render total raw scores. Scores are obtained by summing a child's raw points on each MSEL scale. Raw scores on each subscale are then converted to age‐normed T‐scores (M = 50, SD = 10), based on an original U.S. norming sample. The T‐scores of the fine motor, expressive language, receptive language, and visual reception scales can also be combined to produce the early learning composite (ELC) score (M = 200, SD = 30). In the present study, the ECL was used only as a baseline control. In the original MSEL studies,^12^ the MSEL produced good psychometric properties across the different subscales, with measures of internal consistency ranging from *r* = 0.75 to 0.83, and test‐retest estimates higher than *r* = 0.78 for all scales.

#### Maternal report of child development

As a complement to the MSEL, we asked mothers to complete the short version of the CREDI,^13^ a 20‐item tool that provides a global, overall score capturing children's developmental progress in five domains, including motor, cognitive, language, social–emotional, and mental health. There are different age‐specific versions of the short form CREDI for each of six different age ranges up to 35 months old; in the present study, we used the versions for infants in the 0‐ to 5‐month‐old range (at our 12‐week assessment), 6‐ to 11‐month‐old range (at our 24‐week assessment), and 12‐ to 17‐month‐old range (at our 52‐week assessment). Caregivers respond to each item with “Yes” (scored as 1), “No” (scored as 0), or “I don't know” (scored as 0). All 20 items are summed to create a raw score. Raw scores are then replaced with age‐specific standardized scores based on a multinational comparison study^18^ (available at https://sites.sph.harvard.edu/credi/).

### Statistical analyses

Primary analyses were performed according to the randomized treatment group regardless of compliance (intention‐to‐treat). We used multiple regression models to examine the independent and combined effects of thiamine dosage levels that intervention groups received (i.e., 0, 1.2, 2.4, and 10 mg/daily) on our primary cognitive outcomes at 24 weeks, as well as at 12 and 52 weeks. Additionally, our regression models included a measure of human milk thiamine concentrations at 2 weeks postpartum to control for possible differences in thiamine exposure both prenatally and immediately after birth. Regression models also included the baseline measure of each criterion to control for possible differences at birth in infants’ standing in a specific cognitive domain (e.g., infants’ gross motor level at our baseline assessment at 2 weeks was controlled in separate models that predicted infants’ gross motor levels at 12, 24, and 52 weeks). As such, the inclusion of the autoregressive term meant that our models were examining changes from baseline levels in each of the cognitive domains. Effect sizes are reported as *r* ^2^, and with the results associated with cognitive outcomes at both 24 and 52 weeks, Bonferroni adjustments for multiple tests were applied for these two time points.

Mixed‐effects models with repeated measures were used to test differences in infants’ cognitive performance across time as predicted by treatment dosages of thiamine while also accounting for human milk thiamine concentrations at 2 weeks postpartum. Models included fixed effects for time, group thiamine dosage, time‐by‐group thiamine dosage interaction, and milk total thiamine concentrations at 2 weeks. Polynomial contrasts were used to examine nonlinear aspects of infants’ cognitive development. Separate models were estimated for each of the MSEL outcomes across four assessments (baseline, 12, 24, and 52 weeks) as well as mothers’ reports of infant development on the CREDI across three time points (12, 24, and 52 weeks). A best effect size for mixed effects models with repeated measures is not readily agreed on;^19^ thus, we interpret the produced estimates as these are in the metric of our cognitive outcomes (T‐scores) and highlight meaningful effects in terms of differences in SDs between time points or thiamine dosage levels.

#### Covariates and tests of interactions

A set of theoretically motivated covariates was examined to determine possible inclusion in our primary analysis. The covariates included child sex, maternal and paternal highest level of education, household wealth index, and a standardized measure of infant growth status at 2 weeks postpartum (length‐for‐age Z‐score).

At the time of preregistration,^8^ and based on the cognitive findings of Fattal‐Valevski *et al*.,^1^, ^3^, ^4^ we estimated that we would have power (1 – β error probability) > 0.87 to detect small to medium effect sizes with our secondary outcomes of interest with a planned sample size of *n* = 320, or *n* = 80 per treatment arm.

All analyses were performed in SPSS® Version 25 (2017, IBM Corp, Armonk, NY).

## Results

### Sample characteristics

The trial profile is shown in Figure S1 (online only) and the participant baseline characteristics are shown in Table 1. At baseline, 335 families were participating in the study; usable cognitive data were available for 295 and 309 infants at 24 and 52 weeks, respectively, with migration being the main reason for loss to follow up. At birth, infants’ anthropometric status was typically below WHO Z‐score means in terms of length‐for‐age, a finding that is consistent with other data collected from many LMICs.^10^, ^20^, ^21^ The majority of both the women and their husbands had at least a primary education level. Reflecting the success of randomization, there were no differences among the treatment groups for all characteristics except family wealth. All central analyses initially were run controlling for family wealth, but it was dropped from our presented results because it was a nonsignificant predictor in all models. Table 1 also provides infants’ standardized MSEL scores at 2 weeks. Though these scores were below T‐score norms in the United States of 50 (SD = 10), there were no significant differences in MSEL scores among treatment groups at the 2‐week baseline (*F’*s (3, 331) < 1.49, *P*’s > 0.22).

**Table 1:** Infant, maternal, and household characteristics by treatment group at baseline

	Total (*n = *335)	Placebo (0 mg) (*n* = 83)	1.2 mg (*n = *86)	2.4 mg (*n = *81)	10 mg (*n = *85)
Infants					
Sex, *female*	161 (48%)	43 (52%)	43 (50%)	33 (41%)	42 (49%)
Length‐for‐age (Z‐score) at 2 weeks	−0.62 (1.02)	−0.52 (0.98)	−0.66 (1.11)	−0.69(1.01)	−0.63 (1.01)
MSEL*^a^* at 2 weeks					
Gross motor	36.91 (7.18)	37.60 (6.45)	37.36 (6.97)	35.38 (7.40)	37.21 (7.78)
Fine motor	33.89 (5.96)	34.41 (6.10)	33.79 (5.52)	32.72 (5.84)	34.60 (6.33)
Visual reception	22.97 (3.70)	31.91 (8.11)	31.90 (7.51)	30.38 (8.02)	31.86 (8.73)
Receptive language	31.53 (8.07)	31.91 (8.11)	31.90 (7.51)	30.38 (8.02)	31.86 (8.73)
Expressive language	38.10 (1.36)	38.00 (.93)	38.24 (1.98)	37.88 (.97)	38.26 (1.51)
Mothers					
Age, *years*	28.1 (6.2)	28.3 (6.1)	27.9 (6.7)	28.1 (6.1)	28.1 (5.9)
Parity, *multiparous*	57 (69%)	54 (65%)	54 (63%)	58 (72%)	64 (75%)
Ethnicity, *Khmer*	335 (100%)	83 (100%)	86 (100%)	81 (100%)	85 (100%)
Marital status					
*Married*	330 (98%)	79 (95%)	86 (100%)	81 (100%)	84 (99%)
*Divorced/separated/widowed*	5 (<1%)	4 (5%)	0 (0%)	0 (0%)	1 (1%)
Education					
*None*	40 (12%)	10 (12%)	8 (9%)	13 (16%)	9 (11%)
*Primary (1–6 years)*	161 (48%)	43 (52%)	37 (43%)	40 (49%)	41 (48%)
*Lower secondary (7–9 years)*	83 (25%)	16 (19%)	29 (34%)	19 (24%)	19 (22%)
*Upper secondary (10–12 years)*	43 (13%)	12 (15%)	9 (11%)	8 (10%)	14 (17%)
*Higher education*	8 (2%)	2 (2%)	3 (3%)	1 (1%)	2 (2%)
Milk total thiamine concentrations (μg/L) at 2 weeks	129.1 (74.4)	135.5 (77.7)	129.3 (71.4)	126.3 (77.3)	125.4 (72.3)
Households					
Husband's education					
*None*	38 (11%)	10 (12%)	9 (10%)	9 (11%)	10 (12%)
*Primary (1–6 years)*	151 (45%)	42 (51%)	37 (43%)	39 (48%)	33 (39%)
*Lower secondary (7–9 years)*	97 (29%)	21 (25%)	24 (28%)	23 (28%)	29 (34%)
*Upper secondary (10–12 years)*	34 (10%)	5 (6%)	13 (15%)	8 (10%)	8 (9%)
*Higher education*	15 (4%)	5 (6%)	3 (3%)	2 (3%)	5 (6%)
Household size, *number of people*	3.9 (1.9)	3.7 (1.7)	3.6 (1.8)	4.0 (2.1)	4.1 (2.0)
Median annual household income, *US$ (IQR)*	1620 (950–3500)	1800 (950–3000)	2050 (963–3500)	1600 (1000–3000)	2000 (1200–3500)
Wealth index score*^b^*					
*Poorest*	81 (24%)	22 (27%)	12 (15%)	21 (26%)	25 (29%)
*Second*	69 (21%)	16 (19%)	14 (16%)	20 (25%)	19 (22%)
*Middle*	108 (32%)	26 (31%)	31 (36%)	24 (30%)	27 (32%)
*Fourth*	54 (16%)	14 (17%)	20 (23%)	11 (13%)	9 (11%)
*Wealthiest*	23 (7%)	5 (6%)	8 (10%)	5 (6%)	5 (6%)

Note: Data are mean (SD) or *n* (%), except for household income, which is shown as median (IQR). Percentages may not add up to 100% due to rounding. Appropriate tests were used to test for differences among treatment groups at baseline. With one exception, none of our primary demographic, maternal thiamine, or infant cognitive measures were significantly different (*P’*s *>* 0.05) at 2 weeks. Family wealth index was significantly different among groups, with the 1.2 mg/day group being significantly better off financially (*P* = 0.017).

^*a*^ MSEL, Mullen Scales of Early Learning.

^*b*^ Wealth equity index (WEI) quintiles were calculated based on the Demographic Health Survey Program guidelines (USAID); Cambodian WEI were developed using 2014 DHS data.

### Multiple regression tests of thiamine dosages by intervention groups and human milk total thiamine concentrations

We utilized multiple regression models to investigate possible associations between thiamine dosage levels that intervention groups received (i.e., 0, 1.2, 2.4, and 10 mg/day) and infants’ cognitive outcomes at 12, 24, and 52 weeks, though we highlight 24 weeks as our primary outcome to mark the end of our intervention. Initially, all regression equations included our set of covariates. As none of the covariates were significant predictors of infant cognitive outcomes in our regression models, covariates were not included in the presented models. Regression results are presented in Table 2.

**Table 2:** Thiamine dosage levels and human milk thiamine concentrations predict infant cognitive outcomes at 12, 24, and 52 weeks postpartum

	12 week outcomes					24 week outcomes					52 week outcomes				
Predictors	B	SE B	β	*t*	95% CI	B	SE B	β	*t*	95% CI	B	SE B	β	*t*	95% CI
MSEL*^a^* gross motor															
Milk thiamine at 2 weeks*^b^*	0.00	0.01	0.01	0.17	[−0.01, 0.01]	−0.01	0.01	−0.08	−1.33	[−0.02, 0.00]	−0.01	0.01	−0.04	−0.62	[−0.02, 0.01]
MSEL gross motor at 2 weeks	0.06	0.05	0.06	1.08	[−0.05, 0.17]	**0.19**	**0.06**	**0.19**	3.29^***^	**[0.08, 0.30]**	0.00	0.01	0.001	0.01	[−0.17, 0.17]
Tx thiamine dosage	0.07	0.10	0.04	0.72	[−0.12, 0.27]	0.03	0.11	0.02	0.30	[−0.18, 0.24]	−0.07	0.16	−0.02	−0.42	[−0.38, 0.25}
	*R* ^2^ = 0.01, *F*(3, 306) = 0.56, *P = *0.64					*R* ^2^ = 0.04, *F*(3, 294) = 4.21, *P = *0.01					*R* ^2^ = 0.00, *F*(3, 306) = 0.19, *P = *0.91				
MSEL*^a^* fine motor															
Milk thiamine at 2 weeks*^b^*	0.00	0.01	0.00	0.06	[−0.01, 0.01]	**0.01**	**0.01**	**0.14**	2.48^**^	**[0.00, 0.02]**	−0.01	0.01	−0.08	−1.34	[−0.03, 0.01]
MSEL fine motor at 2 weeks	**0.17**	**0.08**	**0.13**	2.26^*^	**[0.02, 0.32]**	**0.14**	**0.06**	**0.13**	2.27^*^	**[0.02, 0.26]**	**0.22**	**0.11**	**0.10**	2.03^*^	**[**−**0.02, 0.44]**
Tx thiamine dosage	−0.02	0.12	−0.01	−0.15	[−0.25, 0.21]	0.11	0.10	0.07	1.19	[−0.07, 0.30]	0.15	0.17	0.05	0.84	[−0.20, 0.49]
	*R* ^2^ = 0.02, *F*(3, 306) = 1.70, *P = *0.17					*R* ^2^ = 0.04, *F*(3, 294) = 4.38, *P = *0.01					*R* ^2^ = 0.02, *F*(3, 306) = 2.02, *P = *0.08				
MSEL*^a^* visual reception															
Milk thiamine at 2 weeks*^b^*	−0.01	0.01	−0.05	−0.94	[−0.03, 0.01]	0.00	0.01	−0.01	−0.09	[−0.01, 0.01]	0.00	0.01	0.00	0.01	[−0.02, 0.02]
MSEL visual reception at 2 weeks	−0.18	0.16	−0.06	−1.09	[−0.50, 0.14]	0.06	0.11	0.04	0.60	[−0.14, 0.27]	0.14	0.21	0.04	0.65	[−0.28, 0.55]
Tx thiamine dosage	−0.06	0.16	−0.02	−0.40	[−0.37, 0.24]	−0.07	0.10	−0.04	−0.75	[−0.26, 0.12]	−0.16	0.20	−0.05	−0.79	[−0.55, 0.24]
	*R* ^2^ = 0.00, *F*(3, 306) = 0.75, *P = *0.52					*R* ^2^ = 0.00, *F*(3, 294) = 0.47, *P = *0.81					*R* ^2^ = 0.00, *F*(3, 306) = 0.34, *P = *0.79				
MSEL*^a^* receptive language															
Milk thiamine at 2 weeks*^b^*	**0.02**	**0.01**	**0.10**	2.03^*^	**[0.00, 0.03]**	**0.03**	**0.01**	**0.17**	3.04^***^	**[0.01, 0.05]**	0.00	0.01	0.03	0.49	[−0.01, 0.02]
MSEL receptive language at 2 weeks	**0.15**	**0.07**	**0.12**	2.11^*^	**[0.01, 0.29]**	**0.22**	**0.08**	**0.15**	2.70^**^	**[0.06, 0.37]**	0.03	0.06	0.02	0.42	[−0.09, 0.14]
Tx thiamine dosage	0.19	0.15	0.07	1.31	[−0.10, 0.47]	**0.39**	**0.17**	**0.14**	2.54^**^	**[0.09, 0.74]**	−0.04	0.13	−0.02	−0.35	[−0.29, 0.20]
	*R* ^2^ = 0.02, *F*(3, 306) = 3.16, *P = *0.02					*R* ^2^ = 0.07, *F*(3, 294) = 7.88, *P* < 0.00					*R* ^2^ = 0.00, *F*(3, 306) = 0.18, *P = *0.91				
MSEL*^a^* expressive language															
Milk thiamine at 2 weeks*^b^*	−0.00	0.01	−0.03	−0.58	[−0.02, 0.01]	**0.01**	**0.01**	**0.14**	2.45^**^	**[0.00, 0.03]**	0.00	0.01	0.02	0.30	{−0.01, 0.02]
MSEL expressive language at 2 weeks	−0.31	0.33	−0.05	−0.94	[−0.97, 0.34]	−0.44	0.27	−0.09	−1.62	[−0.98, 0.10]	−0.30	0.40	−0.04	−0.74	[−0.49, − 1.09]
Tx thiamine dosage	0.10	0.13	0.05	0.78	[−0.15, 0.35]	**0.19**	**0.10**	**0.11**	2.07^*^	**[0.01, 0.39]**	−0.13	0.14	−0.05	−0.97	[−0.40, 0.14]
	*R* ^2^ = 0.01, *F*(3, 306) = 0.58, *P = *0.63					*R* ^2^ = 0.04, *F*(3, 294) = 3.65, *P = *0.01					*R* ^2^ = 0.01, *F*(3, 306) = 0.50, *P = *0.69				
CREDI*^c^*															
Milk thiamine at 2 weeks*^b^*	0.00	0.00	0.03	0.51	[−0.00, 0.00]	**0.01**	**0.00**	**0.18**	3.11^**^	**[0.00, 0.01]**	**0.01**	**0.00**	**0.13**	2.15^*^	**[0.00, 0.01]**
CREDI or MSEL at baseline*^d^*	0.02	0.01	0.09	1.64	[−0.00, 0.04]	**0.20**	**0.06**	**0.18**	3.18^**^	**[0.08, 0.33]**	**0.20**	**0.08**	**0.14**	2.42^*^	**[0.04, 0.36]**
Tx thiamine dosage	0.01	0.03	0.01	0.23	[−0.05, 0.06]	0.02	0.03	0.03	0.63	[−0.05, 0.08]	0.00	0.04	0.01	0.07	[−0.09, 0.08]
	*R* ^2^ = 0.01, *F*(3, 306) = 1.01, *P = *0.39					*R* ^2^ = 0.07, *F*(3, 294) = 6.79, *P* < 0.00					*R* ^2^ = 0.04, *F*(3, 289) = 3.51, *P = *0.02				

Note: Table results are based on linear regression analyses. Results in the shaded columns associated with 24 weeks are highlighted because 24 weeks was our primary, preregistered outcome time point for the present trial, and corresponded with the end of the thiamine supplementation trial. The 6 months between 24 and 52 weeks coincided with the postintervention period. Significant effects are presented in bold font. SE, standard error.

^*a*^ MSEL, Mullen Scales of Early Learning.

^*b*^ Human milk total thiamine concentrations were calculated as free thiamine + (thiamine monophosphate × 0.871) + (thiamine diphosphate × 0.707).

^*c*^ CREDI, Caregiver Report of Early Development Instrument.

^*d*^ CREDI at 2 weeks was not available; therefore, we used the baseline Mullen ELC score as our autoregressive term when predicting infants’ CREDI scores at 12 weeks in both Model 1 and Model 2. Infants’ CREDI score at 12 weeks was used as the autoregressive terms in models predicting CREDI scores at 24 and 52 weeks.

* *P* < 0.05; ^**^ *P* < 0.01; ^***^ *P* < 0.001.

#### MSEL at 24 weeks

In the regression equations used to predict infants’ cognitive scores on the MSEL at 24 weeks (shaded panel in Table 2), group thiamine dosage levels were significantly and positively predictive of infants’ receptive and expressive language scores. These findings were specific to language development measures; maternal thiamine dosage levels did not show significant associations with other MSEL measures (fine motor, gross motor, and visual reception) when infants were 24 weeks old. Higher milk thiamine concentrations at 2 weeks postpartum were also significantly associated with infants’ more advanced fine motor, receptive language, and expressive language at 24 weeks.

We ran a second set of regression equations in which the group thiamine dosage variable was replaced by a contrast vector testing our preregistered hypothesis that cognitive outcomes at 24 weeks would be significantly stronger for infants whose mothers received 10 mg of thiamine daily than infants whose mothers received 0 mg of thiamine daily. The same pattern emerged, with this contrast not significantly differentiating infants in terms of gross motor, fine motor, and visual reception, whereas both contrasts were significant when predicting receptive and expressive language (Table S1, online only).

Although our achieved power was very limited with our smallest effects (e.g., power <0.30 for visual reception at 24 weeks), we achieved adequate power in both language domains (power >0.80 in the expressive as well as the receptive domains at 24 weeks).

#### MSEL at 12 and 52 weeks

In regression equations predicting MSEL outcomes at 12 weeks, group thiamine dosage levels were not significantly associated with infants’ cognitive scores in any of the MSEL domains. In the language domain, milk total thiamine concentration at 2 weeks was significantly and independently associated with infants’ receptive language level at 12 weeks; specifically, higher concentrations of thiamine in human milk at 2 weeks heralded greater receptive language ability at 12 weeks.

In models predicting cognitive outcomes at 52 months (hence 6 months after the end of the intervention trial), the variable representing group thiamine dosage levels was no longer predictive of any MSEL outcome. Likewise, human milk thiamine concentrations 2 weeks were no longer predictive of outcomes at 52 weeks.

#### CREDI

Similar regression models were used to examine associations between group thiamine dosage levels and milk thiamine concentrations at 2 weeks as predictors, and maternal reports of children's development on the CREDI at 12, 24, and 52 weeks postpartum. Because we did not collect the CREDI at our 2‐week baseline, we used the MSEL's ELC score at 2 weeks as our baseline control when predicting CREDI scores at 12 weeks. We used the 12‐week CREDI score as the control in models used to predict 24‐ and 52‐week CREDI outcomes. The variable representing group thiamine dosage levels was not significantly predictive of CREDI scores at any age (Table 2). However, milk thiamine concentration at 2 weeks was associated with significantly higher CREDI scores at both 24 and 52 weeks.

### Infant cognitive development across the first year of life

Mixed‐effects models with repeated measures were used to explore differences in infants’ cognitive development trajectories across time as predicted by treatment group thiamine dosage level.

#### MSEL: gross motor, fine motor, and visual reception domains

**Figure 1:**
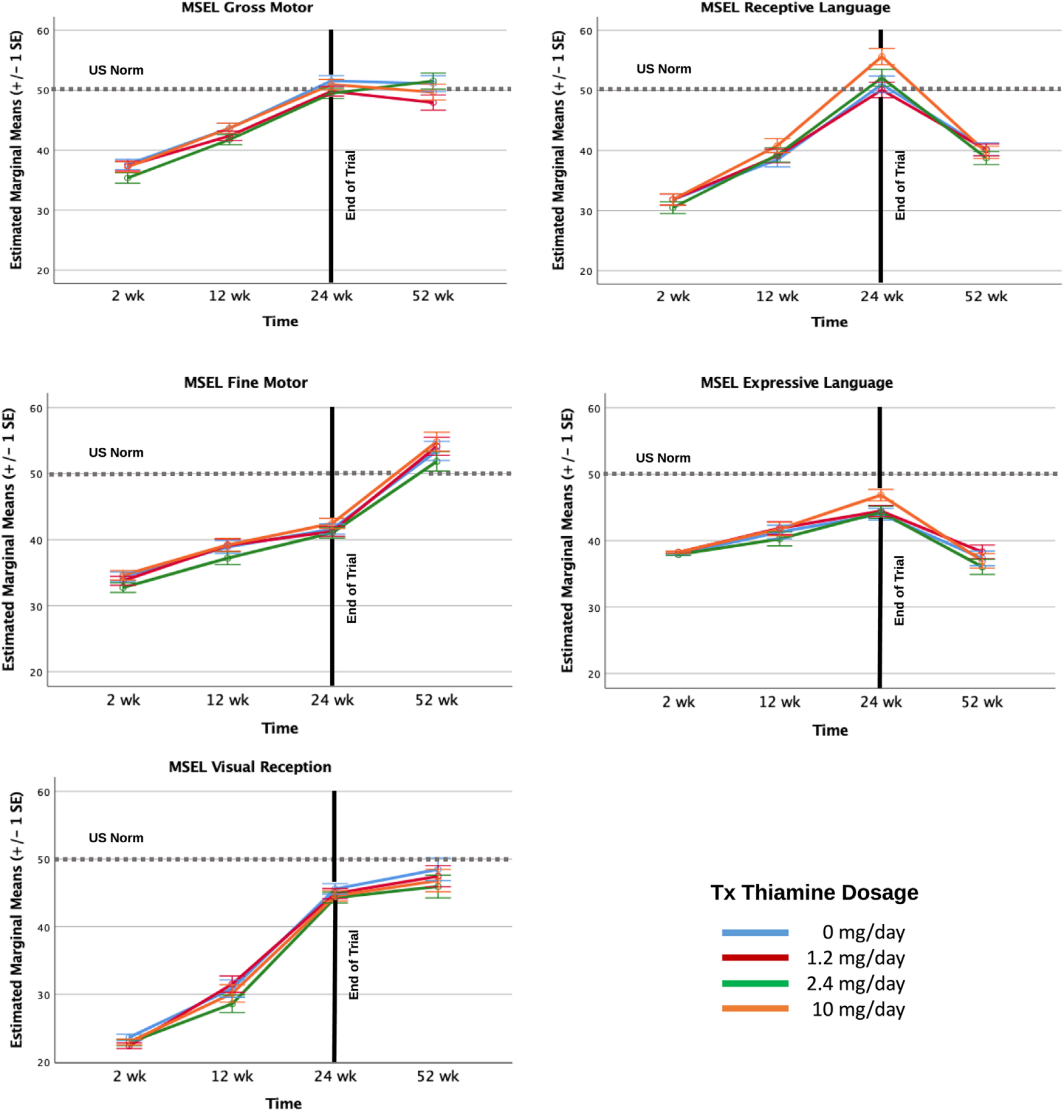
Developmental trajectories of MSEL change by thiamine dosage levels.

As shown in Table 3, and consistent with findings stemming from our regression models reported above, thiamine supplementation dosage did not significantly account for differences in infants’ cognitive development across weeks 2 through 52 in the gross motor, fine motor, and visual reception domains (*F’*s (1, 324) *<* 1.04*, P’*s > 0.31). Similarly, the time‐by‐thiamine supplementation level interactions were not significant in these three models either (*F’*s (3, 748) *<* 0.42*, P’*s *>* 0.74). The effect of time, however, was significant in all three models (*F’*s (3, 659) *>* 116.85*, P’*s *<* 0.001), with estimates indicating significant developmental progress between 2 and 52 weeks, 12 and 52 weeks in all three domains, and significant positive development between 24 and 52 weeks in the fine motor and visual reception domains, but not the gross motor domain, where the change was nonsignificant. Inspection of the polynomial contrasts revealed significant linear effects for fine motor and visual reception development (Estimates = 60.59 (SE = 2.64) and 90.36 (SE = 3.01), *P’*s < 0.001, respectively) and a significant linear and quadratic effect in infant gross motor development (Estimate = 48.33 (SE = 2.58) and Estimate = −5.56 (SE = 1.17), *P’*s < 0.001, respectively), as shown by the leveling off of gross motor development between 24 and 52 weeks (Fig. 1). Milk thiamine concentrations were not significantly associated with infant cognitive development in these three domains between 2 and 52 weeks. Finally, as shown in Figure 1, although infants were well below U.S. norms at 2 weeks postpartum, by 52 weeks, infants’ standardized scores in both motor domains were at or just higher than U.S. norms, and less than a half a SD below U.S. norms in the visual reception domain.

**Table 3:** Coefficient estimates from a nonlinear mixed‐effects model with repeated measures predicting infant cognitive development between 2 and 52 weeks

	MSEL*^a^* gross motor				MSEL*^a^* fine motor				MSEL*^a^* visual reception				MSEL*^a^* receptive language				MSEL*^a^* expressive language			
	Est	SE	*t*	*P*	Est	SE	*t*	*P*	Est	SE	*t*	*P*	Est	SE	*t*	*P*	Est	SE	*t*	*P*
Intercept	**50.73**	**0.80**	**63.24**	**0.00**	**52.92**	**0.81**	**64.96**	**0.00**	**48.51**	**0.89**	**54.54**	**0.00**	**38.35**	**0.91**	**42.11**	**0.00**	**37.48**	**0.68**	**55.18**	**0.00**
Milk thiamine at 2 weeks	−0.00	0.00	−0.67	0.50	0.00	0.00	0.75	0.46	−0.00	0.00	−0.62	0.54	**0.01**	**0.00**	**2.95**	**0.00**	**0.01**	**0.00**	**2.87**	**0.01**
Time																				
2–52 weeks	**13.52**	**0.83**	**16.14**	**0.00**	**19.32**	**0.84**	**22.86**	**0.00**	**25.21**	**0.94**	**26.81**	**0.00**	**8.53**	**0.96**	**8.89**	**0.00**	−0.21	0.74	−0.28	0.77
12–52 weeks	**7.89**	**0.84**	−**9.31**	**0.00**	**14.56**	**0.86**	**16.94**	**0.00**	**17.60**	**0.96**	**18.37**	**0.00**	1.07	0.97	1.10	0.27	**3.00**	**0.75**	**4.00**	**0.00**
24–52 weeks	0.05	0.81	−0.07	0.95	**11.93**	**0.83**	**14.21**	**0.00**	**2.92**	**0.99**	**2.95**	**0.00**	−**10.78**	**0.95**	**11.40**	**0.00**	−**6.31**	**0.72**	−**8.82**	**0.00**
Tx thiamine dosage*^b^*	−0.07	0.12	−0.61	0.54	0.16	0.12	1.26	0.21	−0.16	0.13	−1.17	0.24	−0.03	0.14	−0.24	0.81	−0.12	0.10	−1.16	0.25
Time ^*^ Tx thiamine dosage																				
2–52 weeks ^*^ dosage level	0.09	0.16	0.55	0.58	0.12	0.16	0.77	0.45	0.16	0.18	0.90	0.37	0.01	0.18	0.08	0.94	0.14	0.14	1.04	0.30
12–52 weeks ^*^ dosage level	0.15	0.16	0.89	0.37	0.17	0.17	1.01	0.31	0.09	0.18	0.47	0.64	0.22	0.18	1.22	0.23	0.21	0.14	1.47	0.14
24–52 weeks ^*^ dosage level	0.09	0.16	0.57	0.57	0.04	0.16	0.26	0.80	0.08	0.19	0.44	0.66	**0.43**	**0.18**	**2.37**	**0.02**	**0.30**	**0.13**	**2.13**	**0.03**

Note: Table results are based on nonlinear mixed‐effects models with repeated measures. Significant effects are presented in bold font.

^*a*^ MSEL, Mullen Scales of Early Learning. All MSEL scores are standardized T‐scores, with a mean of 50 and SD of 10.

^*b*^ Tx thiamine dosage = thiamine supplementation dosages used in treatment trial (0, 1.2, 2.4, and 10 mg/daily) between 2 and 24 weeks.

#### MSEL: receptive and expressive language domains

Results in both language domains were markedly different (Table 3). The overall effect of time was significant in both language domains (*F’*s (3, 659) *>* 33.2*, P’*s *<* 0.001), but, as shown in Table 3 and Figure 1, infants’ standardized language scores declined between 24 and 52 weeks. Polynomial contrasts highlighted that there was a significant positive linear trend across the full 2‐ to 52‐week period in the receptive language domain (Estimate = 37.41 (SE = 2.97), *t* = 12.61, *P* < 0.001) but not the expressive language domain (Estimate = 2.62 (SE = 2.27), *t* = 1.15, *P* = 0.25), where standardized scores at 2 and 52 weeks were similar. A significant and stronger nonlinear effect highlighted a significant decline in infants’ standardized scores in both language domains between 24 and 52 weeks (Estimate = −18.23 (SE = 1.34) *t* = −13.57, *P* < 0.00 and Estimate = −9.13 (SE = 1.02), *t = *−8.90 *P*’s < 0.001, respectively). As shown in Figure 1, relative to U.S norms, infants in this study reached comparable language scores by 24 weeks, but at 52 weeks scored approximately 1 SD lower than U.S. infants who were used to norm the MSEL's language domains.

Next, although thiamine supplementation levels accounted for marginally significant growth in infants’ receptive language development across the entire 2‐ through 52‐week period (*F* (1,329)* = *3.18*, P = *0.06), the time‐by‐thiamine dosage level interaction accounted for significant receptive language development during this period (*F* (3,726)* = *2.86*, P = *0.04). This interaction effect was largely accounted for by the time‐by‐thiamine dosage level between 24 and 52 weeks, which showed a steeper decline in the receptive language scores of infants of mothers receiving 10 mg/day relative to declines at the other supplementation levels.

As indicated by infants’ expressive language development across the entire 2 through 52 weeks, thiamine supplementation levels were not significantly associated with infant expressive language development (*F* (1, 329)* = *0.55*, P = *0.46). However, the time‐by‐thiamine dosage level interaction was marginally associated with expressive language development during this period (*F’*s (3, 726)* = *2.38*, P = *0.06), but there was a significant time‐by‐dosage interaction between 24 and 52 weeks. As shown in Table 3 and Figure 1, infants whose mothers received 10 mg of thiamine daily scored best in both language domains by the end of the trial period at 24 weeks, but they also showed the steepest subsequent decline in standardized language scores relative to other supplementation levels.

Finally, as shown in Table 3, higher milk total thiamine concentrations at 2 weeks postpartum were associated with significant growth in both language domains across the 2‐ to 52‐week period of development.

#### CREDI

**Figure 2:**
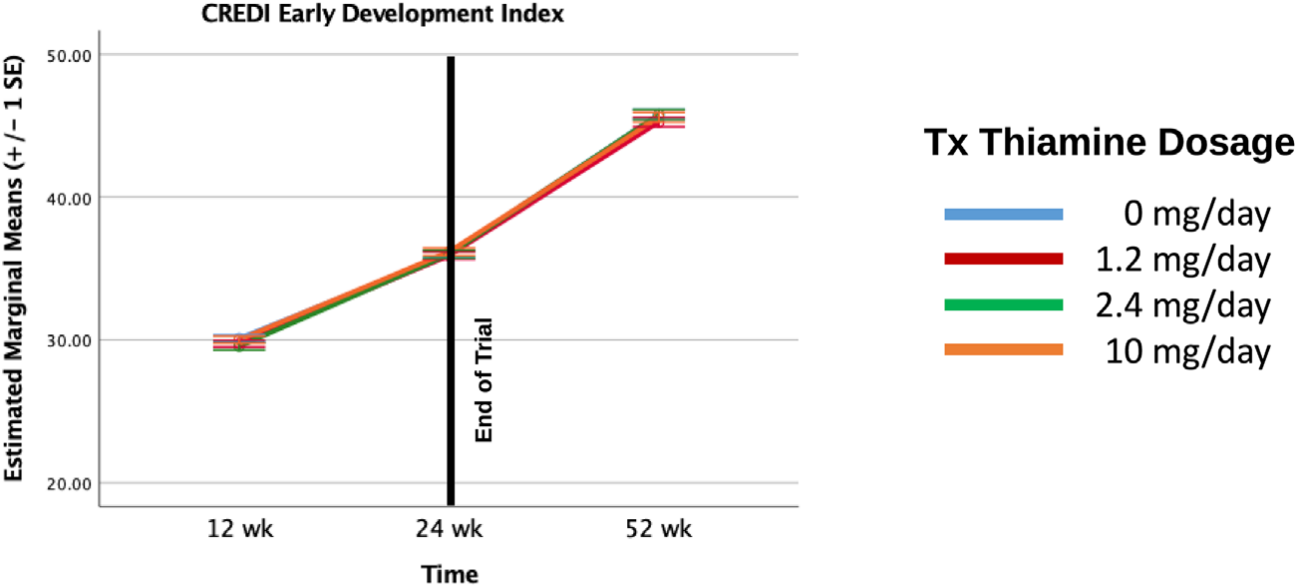
Developmental trajectories of CREDI change by thiamine dosage levels.

The same mixed effects model with repeated measures was used to examine maternal reports of infant development on the CREDI. In contrast to the MSEL scores, we could only model maternal reports on the CREDI between 12 and 52 weeks. As shown in Table 4, we did not find a significant effect of thiamine supplementation level nor was there a significant time‐by‐thiamine dosage level interaction. We did, however, find a significant linear effect of time, indicating significant early development across the 12‐ to 52‐week period as reported by mothers (Fig. 2). Also, there was a significant positive main effect of milk total thiamine concentrations such that higher exposure to thiamine prenatally and immediately following birth (proxy measure: 2‐week milk thiamine concentrations) was associated with infants showing significantly more advanced general development between 12 and 52 weeks.

**Table 4:** Coefficient estimates from a nonlinear mixed‐effects model with repeated measures predicting Caregiver Reports of Infant Early Development (CREDI) between 12 and 52 weeks

	CREDI*^a^*			
	Est	SE	*t*	*P*
Intercept	**45.23**	**0.25**	**3036.03**	**0.00**
Milk thiamine at 2 weeks	**0.02**	**0.00**	**3.14**	**0.00**
Time				
12–52 weeks	**15.93**	**0.24**	**65.95**	**0.00**
24–52 weeks	**9.76**	**0.23**	**42.45**	**0.00**
Tx thiamine dosage*^b^*	−0.01	0.03	−0.23	0.82
Time ^*^ Tx thimaine dosage				
12–52 weeks ^*^ dosage level	0.02	0.05	0.39	0.70
24–52 weeks ^*^ dosage level	0.03	0.04	0.63	0.53

Note: Table results based on nonlinear mixed‐effects models with repeated measures. Significant effects are presented in bold font.

^*a*^ CREDI, Caregiver Report of Early Development Index.

^*b*^ Tx thiamine dosage = thiamine supplementation dosages used in treatment trial (0, 1.2, 2.4, and 10 mg/daily).

## Discussion

We supplemented lactating mothers in Cambodia with four different thiamine dosages (0, 1.2, 2.4, and 10 mg/day) to investigate possible differences in infants’ cognitive development at select points during the first year of life. Several sets of findings in the present study provided confirmation of previous evidence that thiamine status is important to infants’ neurocognitive development.

### Positive association between thiamine dosage levels and infant language development

Most important, the level of thiamine that infants’ lactating mothers received in a daily supplement beginning at 2 weeks postpartum displayed a dose–response relationship to infants’ MSEL receptive and expressive language development at 24 weeks, which marked the end of our supplementation trial. Notably, infants whose mothers received a 10 mg daily thiamine supplement displayed significantly enhanced performance on both MSEL language measures at 24 weeks relative to all other supplementation levels (and, in particular, the 0 mg daily group). These findings indicate that thiamine supplementation at 10 mg daily for mothers at risk of thiamine deficiency confers a significant degree of protection for their infants’ language development during this early period of life. Interestingly, thiamine biomarker status indicative of sufficient status was achieved at lower levels of supplementation (1.2 mg/day), as reported by our own colleagues.^14^ However, levels closer to 10 mg/day appear necessary to achieve language progress that brings infants from LMICs in line with norms for 6‐month‐olds in more advantaged countries.

Human milk total thiamine concentrations measured at the 2‐week, preintervention time point provided another important source of evidence regarding the importance of adequate thiamine for infants’ neurocognitive development. Specifically, infants whose mothers showed higher milk thiamine concentrations at 2 weeks (our estimate of infants’ access to thiamine during the prenatal and early postnatal periods) displayed more advanced cognitive outcomes at 24 weeks. This pattern of positive association emerged for both infants’ MSEL scores (receptive and expressive language and fine motor scores) and their maternally reported CREDI scores, suggesting that infants’ perinatal access to adequate thiamine may have relatively broad implications for neurocognitive development.

### Cognitive performance declined postintervention

Analyses focusing on developmental trajectories revealed that the rate at which infants made progress in MSEL‐measured language domains slowed significantly during the 6‐month period after maternal thiamine supplementation ceased (24–52 weeks). Moreover, infants whose mothers had received higher doses of thiamine in previous months tended to display more precipitous rates of decline in language development once supplementation ended, with all protection conferred by thiamine supplementation during the first 6 months nullified by 52 weeks. One possible perspective on these findings is that they provide yet further indication that infants’ developing language systems require adequate thiamine. Interestingly, the pattern of cognitive decline was specific to the language domain; other domains of cognitive function, such as the MSEL measures of gross motor, fine motor, and visual reception abilities, displayed trajectories of sustained developmental progress beyond the thiamine supplementation period. This pattern could possibly be interpreted to underscore how sensitive language development may be to thiamine levels during the early months of life.^4^, ^11^ Of course, the fall‐off in trajectory of language development that infants displayed from 24 to 52 weeks must be interpreted with an abundance of caution, as the conclusion of our thiamine intervention at 6 months coincides with the age at which complementary foods are introduced to infants.^22^ Other nutrients, particularly iron, are known to play a vital role in neurocognitive development and to also be lacking in complementary foods in LMICs,^23^ which could confound our findings at 52 weeks. Infant participants in this study had similar diet quality and quantity to those surveyed in the most recent Cambodian Demographic and Health Survey.^6^ One third of infants (35%) had an acceptable minimum dietary diversity score (4 or more food groups), 88% were still breastfed, and 81% met the minimum meal frequency (3 or more meals), as compared with 36%, 80%, and 68%, respectively, among children 9–12 months in the 2014 CDHS. In sum, it is not yet possible to draw clear conclusions regarding the precise implications of infants’ declining trajectory in language development between the conclusion of the intervention at 24 weeks to the 52‐week postintervention time point. Further investigation of this important issue is clearly warranted.

### Comparative longitudinal cognitive trajectories in relation to thiamine

Infants showed significant developmental gains in their gross motor, fine motor, and visual reception capacities across the first year of life as measured by the MSEL. It is worth noting that, relative to infants in the original North American norming sample,^12^ the Cambodian infants were more than one standard deviation below the T‐score mean of 50 on both domains of motor control at 2 weeks postpartum, and more than two standard deviations lower than U.S. means in visual reception. However, by 52 weeks, their scores approximated North American norms in these domains, suggesting that the Cambodian sample may have overcome, at least temporarily, some of the developmental risks typical of LMICs. Improvements in postural control as well as enhanced visual processing and accompanying fine‐motor dexterity develop early and rapidly in life^24^ and may have benefited from cultural affordances typical of warmer climates such as Cambodia, including less encumbering clothing, open floor plans with easy passage outdoors, and caregivers who encourage physical exploration earlier than Western caregivers.^25^ Despite these gains and contrary to prediction, we generally did not see differences in the rate or magnitude of motor control and visual reception gains among the intervention groups.

A different picture emerged with infants’ language development. Infants of mothers in all thiamine supplementation groups showed a significant decrease in their standardized language scores between 24 and 52 weeks, suggestive of slowing in language development relative to U.S. norms. Our analyses indicated that higher levels of maternal thiamine supplementation—specifically, 10 mg daily thiamine—were associated with especially steep declines in the trajectory of language development in the 24‐ to 52‐week period. It is important to recognize that the magnitude of this developmental slowing observed across our sample was not trivial, as infants decreased approximately 1.5 standard deviations from their 24‐week standardized score and were approximately a full standard deviation below U.S. norms by 52 weeks. These results are consistent with studies that report a widening gap in cognitive test scores over time between children from high‐ versus low‐risk contexts,^26^, ^27^ as well as a decline in scores among children specifically from LMICs.^28^ In a study of Gambian infants, Milosavljevic and colleagues^29^ found no differences between the standardized MSEL scores of infants in their study and U.S. norms when infants were between 5 and 9 months. However, by 10–14 months, and more strongly so by 20–24 months, Gambian infants scored approximately 1.5 standard deviations lower than U.S. norms.^28^ Similarly, Jensen and colleagues^30^ reported a normal range of cognitive scores on the MSEL in a sample of 6‐month‐old Bangladeshi infants. However, they saw a greater than 2 SD decline in standardized MSEL scores between 6 and 27 months, with risk factors such as poverty, family care, and immune functioning strongly associated with declines in receptive and expressive language scores.^30^ Although we did not see declines by 12 months in motor control or visual reception in the present study, these other studies raise the possibility that declines in these domains may emerge somewhat later or require a greater accumulation of risk exposure before development is noticeably affected. Our pattern of results in both language domains is in line with longitudinal studies in other LMICs and highlights the importance of early life as a critical period for language development.^11^

Additionally, in contrast to infants’ language scores on the MSEL, which declined relative to U.S. norms after 24 weeks, infants’ early development, as measured by maternal report on the CREDI, continued to increase significantly between 24 and 52 weeks. Like infants’ cognitive scores on the MSEL, mother's reports of greater infant development on the CREDI were associated with higher milk thiamine concentrations at 2 weeks. Here too, a parental report of early infant development seems to have been sensitive to the effects of perinatal thiamine. Both the MSEL and CREDI findings further substantiate growing recognition of the critical role that prenatal nutrition plays in early brain development that is foundational to postnatal cognitive development. However, one might ask why mothers in our sample seemed to have been less aware of the slowing in their infants’ developmental trajectories between 24 and 52 weeks than the MSEL language scores captured. On the one hand, it might be that the MSEL is more sensitive to children's capacities as they get older,^31^ thus better than parents at detecting age‐related declines in children's performance. To the extent that an absence of supplemental maternal thiamine in the 6‐month period between 24 and 52 weeks played a genuine role in the slowing of children's developmental trajectory, a performance‐based test such as the MSEL may prove more sensitive than parental reports. Alternatively, the decline in children's standardized MSEL scores, specifically their language development scores, was relative to U.S. norms, whereas the CREDI was normed largely with infants from multiple LMICs.^17^ Quite likely, the mothers in our sample were accurately reporting on gains in their infants’ developmental capacities across the first year of life, even if these gains may have been slowing and apparently decreasing relative to children from more resourced countries. As the decrease in development appears to be generalized in the population, these slower trajectories likely appear typical to care‐givers.

### Limitations and questions

#### Nature of the evidence

As we have described, this study produced two major strands of evidence that adequate thiamine is important to infants’ developing language capacities. The double‐blind randomized controlled trial design within which these findings emerged justifies interpreting the findings in causal terms. That said, measurement of mothers’ 2‐week milk thiamine concentrations occurred prior to randomized thiamine supplementation, meaning that results linking mothers’ 2‐week milk thiamine concentrations to infants’ subsequent cognitive outcomes are correlational. Caution is thus warranted regarding causal inference with respect to these findings. Clearly, however, the findings increase the plausibility of the hypothesis that infants’ prenatal access to adequate thiamine matters for their subsequent neurocognitive thriving, particularly in the language domain.

Taking a strongly skeptical stance toward the findings reported here, one might be tempted to argue that signs of thiamine effects on infants’ neurocognitive development were sparse—emerging primarily at the 24‐week time point and primarily in the language domain alone—and thus might be spurious. Additionally, because factors such as early cognitive stimulation were not included in the current analyses, potentially important environmental factors cannot be ruled out. Nevertheless, we firmly question the inclination to discount these results. Previous findings^1^, ^3^ highlighted language development as a domain particularly vulnerable to infantile thiamine deficiency. Our findings, from an appropriately powered, double‐blind, randomized controlled trial, clearly documented benefits of 10 mg/day thiamine supplementation specifically for both receptive and expressive language at 24 months, a preregistered prediction (achieved power of >0.80 in both language domains). Moreover, our measure of milk thiamine concentrations at the 2‐week, preintervention time point revealed additional evidence of positive associations between maternal thiamine status and infants’ 24‐week cognitive progress in language as well as other developing domains. These findings, thus, offer an important contribution to available evidence indicating the importance of thiamine for infants’ neurocognitive thriving. That said, numerous important questions remain, to be considered, as described below.

#### Little relation between thiamine dosage and development in nonlanguage domains

Contrary to expectation, significant associations generally were not detected between levels of maternal thiamine supplementation and infant motor development or visual reception. Moreover, by 24 weeks—and even more so by 52 weeks—infants in the present study achieved motor and visual scores on the MSEL that were comparable to U.S. norms. Harel and colleagues^32^ found that infants fed a thiamine‐deficient formula for over a month‐long period during the first 2 years of life exhibited movement and motor skill difficulties by age 5 relative to dietarily healthy preschoolers. Subsequent neuroimaging of these thiamine‐deficient infants implicated the cerebellum and basal ganglia, both of which are known to assist in postural control and balance.^1^, ^33^ Ocular and visual impairments also have been noted in the case of thiamine deficiency.^34^ Although infants in the present study did not show clinical symptoms of thiamine deficiency,^7^ it is possible that motor and visual reception delays or deficits might emerge in the second year of life and beyond, as has been shown more generally in LMICs contending with malnutrition.^28^ Another, nonmutually exclusive, possibility is that something about developing language systems is especially vulnerable to thiamine deficiency. Here, we point to the vital role that thiamine likely plays in metabolic energy and neurosynthesis.^4^ Language learning during the highly critical infancy period,^11^ with attendant assembly of key neural circuits central to language processing, may place a high demand on just these resources. All in all, accumulating evidence confirms the importance of thiamine to developmental progress in language capacities during infancy and beyond. The extent to which other developing systems are affected remains in question, and the precise metabolic and developmental pathways at play require further investigation.

#### Supplementation duration

On the one hand, positive associations between 2‐week maternal thiamine concentrations and some MSEL‐ and CREDI‐based measures of infants’ cognitive progress at 12, 24, and 52 weeks strengthen the hypothesis that infants’ prenatal access to thiamine matters for neurocognitive development. On the other hand, the apparent decline in infants’ progress in language between 24 and 52 weeks postpartum—after supplementation ceased—potentially strengthens the hypothesis that the protective benefit to infants’ neurocognitive development will be greater if thiamine supplementation continues longer. Many specific questions arise regarding supplementation duration, such as: Why did maternal thiamine supplementation dosage not have an effect on infants’ cognitive development on any measure at 12 weeks, yet it did display effects on language development at 24 weeks? Why were 2‐week milk thiamine concentrations positively associated with a broader range of cognitive outcomes than emerged in analyses probing positive effects of maternal thiamine supplementation? Why did infants whose mothers received higher dosages of thiamine supplementation display steeper fall‐offs in language development progress, when higher supplementation dosages might have instead mitigated the postsupplemention rate of decline in language development? Rather than attempt to offer highly provisional answers to such important questions, we instead note that another clear implication of our full collection of findings is the need for a larger trial in which multiple cognitive outcomes are again investigated, and thiamine supplementation not only includes (and possibly precedes) pregnancy but also continues beyond infants’ first 12 months of life.

## Conclusions

Our findings provide the first experimental evidence that thiamine supplementation among lactating mothers at risk of thiamine deficiency protects their infants’ neurocognitive development, with particular benefit to developing language capacities. Daily maternal thiamine supplementation of 10 mg, a dose well above the RDA, seems to be particularly efficacious in this regard relative to no supplementation and dosages of 1.2 and 2.4 mg daily. Results from the study also reveal that an estimate of mothers’ presupplementation thiamine status presages infants’ subsequent neurocognitive functioning. This finding provides preliminary evidence that infants’ neurocognitive development may benefit most if maternal thiamine supplementation begins prenatally. Lastly, infants’ thiamine‐related developmental progress unfortunately displayed steep declines when compared with U.S. norms after maternal thiamine supplementation ceased at 6 months of age. Maternal thiamine supplementation and/or integration of nutrient‐rich complementary foods well beyond infants’ first 6 months may be necessary to sustain neurocognitive gains. Important questions remain, particularly with respect to the appropriate duration of thiamine supplementation and/or fortification programs during pregnancy, infancy, and beyond. In any case, our findings further confirm that adequate thiamine intake matters for infants’ neurocognitive thriving.

## Author contributions

J.R.M. and D.A.B. drafted the manuscript. K.C.W., K.H, T.J.G., F.T.W., J.R.M., and D.A.B. conceived the study and wrote the initial study protocol. M.B., S.P., K.C., and J.G. assisted in developing the protocol. H.K., M.B., and S.P. facilitated implementation of the study. J.G. and K.C. were involved in study coordination. D.H., S.S.‐F., and L.H.A. performed human milk thiamine analyses. J.R.M. and D.A.B. performed statistical analyses. J.R.M. accepts responsibility for the integrity of the data analyzed. All authors participated in, read, and approved the final manuscript.

## Competing interests

The authors declare no competing interests.

## Supporting information


**Figure S1**. Trial profile and participant flow.

**Table S1**. Thiamine dosage contrast (0 mg versus 10 g daily), human milk thiamine concentrations predict infant cognitive outcomes at 24 weeks postpartum.
