# Supplementary material for: Thiamine supplementation holds neurocognitive benefits for breastfed infants during the first year of life
Source: Ann N Y Acad Sci. 2021 Jun 7;1498(1):116–32. doi: 10.1111/nyas.14610 (PMC9291201; doi:10.1111/nyas.14610)
Supplement: Supplementary file 1 — Figure S1. Trial profile and participant flow. [file NYAS-1498-116-s002.docx]

included in secondary cognitive

analyses

77

77 Had adequate data for analysis of primary outcome

2 Did not have adequate data for analysis of primary outcome

1 No longer breastfeeding; infant

formula-fed

1 Migration mother only


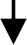


included in secondary cognitive

analyses

72

Had adequate data for analysis of

primary outcome

Did not have adequate data for analysis of primary outcome

72

0

9 Discontinued intervention

2 Withdrew consent

4 Migration (entire family)

1 Visiting relatives for extended period 1 Took family member to hospital out of

province 1 Mother ill

included in secondary cognitive

analyses

73

73 Had adequate data for analysis of primary outcome

1 Did not have adequate data for analysis of primary outcome

1 No longer breastfeeding; infant

formula-fed

**335 mothers randomised**

**181 Mothers excluded**

*166 Did not meet inclusion criteria*

2 Mother not aged between 18-45 years 11 Pregnancy was abnormal

21 Did not intend to exclusively breastfeed for 6 months

39 Did not reside in Kampong Thom province, or was planning to move

84 Participating in nutrition programs beyond normal care 8 Not willing for biological samples to be collected

1 Another woman in the same household enrolled in study

*15 declined to participate (reasons)*

*5* Infant was ill

1. Mother ill, was in an accident 2 Husband did not consent
2. Caesarean delivery

*5* No reason provided

**516 mothers assessed for eligibility**

83 Assigned to 0 mg group

86 Assigned to 1.2 mg group

81 Assigned to 2.4 mg group

85 Assigned to 10 mg group

included in secondary cognitive

analyses

73

Had adequate data for analysis of

primary outcome

Did not have adequate data for analysis of primary outcome

73

0

10 Discontinued intervention

2 Withdrew consent

7 Migration (entire family) 1 Infant death

7 Discontinued intervention

1 Withdrew consent

3 Migration (entire family)

2 Visiting relatives for extended period 1 No reason provided

11 Discontinued intervention

2 Withdrew consent

7 Migration (entire family)

1 Took infant to hospital out of province 1 Another woman enrolled in study

moved into same household

**Supplemental Figure S1.** Trial profile and participant flow.
