# Supplementary material for: Thiamine supplementation holds neurocognitive benefits for breastfed infants during the first year of life
Source: Ann N Y Acad Sci. 2021 Jun 7;1498(1):116–32. doi: 10.1111/nyas.14610 (PMC9291201; doi:10.1111/nyas.14610)
Supplement: Supplementary file 2 — Table S1. Thiamine dosage contrast (0 mg versus 10 g daily), human milk thiamine concentrations predict infant cognitive outcomes at 24 weeks postpartum. [file NYAS-1498-116-s001.docx]

***Supplemental Table S1.*** ***Thiamine Dosage Contrast (0mg vs. 10mg daily), Human Milk Thiamine Concentrations Predict Infant Cognitive Outcomes at 24 Weeks Postpartum***

|  |  | **24 Week Outcomes** | | | | | |
| --- | --- | --- | --- | --- | --- | --- | --- |
| **PREDICTORS** |  | **B** | **SE B** | $\boldsymbol{\beta}$ | ***t*** | **95% CI** |  |
| MSEL^1^ Gross Motor |  |  |  |  |  |  |  |
| Milk thiamine at 2 weeks^*^ |  | -.01 | .01 | -.08 | -1.33 | [-.02, .00] |  |
| MSEL Gross Motor at 2 weeks |  | **.19** | **.06** | **.19** | **3.28***** | **[.08, .30]** |  |
| Thiamine Contrast (0mg vs. 10mg) |  | -.18 | .59 | -.02 | -.31 | [-1.35, .98] |  |
|  |  | *R*^2^ = .04, *F*(3, 294) = 4.24, *p* = .01 | | | | | |
| MSEL^1^ Fine Motor |  |  |  |  |  |  |  |
| Milk thiamine at 2 weeks^*^ |  | **.01** | **.01** | **.14** | **2.48**** | **[.00, .02]** |  |
| MSLE Fine Motor at 2 weeks |  | **.14** | **.06** | **.13** | **2.27*** | **[.01, .25]** |  |
| Thiamine Contrast (0mg vs. 10mg) |  | .51 | .53 | .05 | 1.00 | [-.52, 1.57] |  |
|  |  | *R*^2^ = .04, *F*(3, 294) = 4.12, *p* = .01 | | | | | |
| MSEL^1^ Visual Reception |  |  |  |  |  |  |  |
| Milk thiamine at 2 weeks^*^ |  | .00 | .01 | -.01 | -.09 | [-.01, .01] |  |
| MSEL Visual Reception at 2 weeks |  | .06 | .11 | .03 | .55 | [-.15, .26] |  |
| Thiamine Contrast (0mg vs. 10mg) |  | -.52 | .54 | -.06 | -.96 | [-1.59, .54] |  |
|  |  | *R*^2^ = .00, *F(*32, 294) = 0.43, *p* = .73 | | | | | |
| MSEL^1^ Receptive Language |  |  |  |  |  |  |  |
| Milk thiamine at 2 weeks^*^ |  | **.03** | **.01** | **.17** | **3.03***** | **[.01, .05]** |  |
| MSELReceptive Language at 2 weeks |  | **.22** | **.08** | **.15** | **2.70**** | **[.06, .37]** |  |
| Thiamine Contrast (0mg vs. 10mg) |  | **1.80** | **.92** | **.11** | **1.98*** | **[.09, 3.61]** |  |
|  |  | *R*^2^ =.07, *F*(3, 294) = 6.96, *p* < .00 | | | | | |
| MSEL^1^ Expressive Language |  |  |  |  |  |  |  |
| Milk thiamine at 2 weeks^*^ |  | **.01** | **.01** | **.14** | **2.47**** | **[.00, .03]** |  |
| MSEL Expressive Language at 2 weeks |  | -.44 | .27 | -.09 | -1.63 | [-.98, .09] |  |
| Thiamine Contrast (0mg vs. 10mg) |  | **.96** | **.56** | **.12** | **2.01*** | **[.11, 2.06]** |  |
|  |  | *R*^2^ = .04, *F*(3, 294) = 3.69, *p* = .02 | | | | | |

*Note.* Table results based on linear regression models.
